# Serum Ferritin in Metabolic Syndrome—Mechanisms and Clinical Applications

**DOI:** 10.3390/pathophysiology29020023

**Published:** 2022-06-17

**Authors:** Shrey Kumar Srivastav, Irfan Ahmad Mir, Naman Bansal, Pankaj Kumar Singh, Rashmi Kumari, Ajoy Deshmukh

**Affiliations:** 1 Department of Medicine, School of Medical Science and Research, Sharda University, Uttar Pradesh 201306, India; shrey.srivastav@sharda.ac.in (S.K.S.); naman.bansal@sharda.ac.in (N.B.); rash.kumari2407@gmail.com (R.K.); hanamir765742@gmail.com (A.D.); 2 Department of Emergency Medicine, School of Medical Science and Research, Sharda University, Uttar Pradesh 201306, India; pankajmbbsru@gmail.com

**Keywords:** serum ferritin, metabolic syndrome, endocrinology, marker

## Abstract

Metabolic syndrome (MS) is a cluster of conditions including central obesity, hypertriglyceridemia, low HDL cholesterol, hyperglycaemia, and hypertension with a prevalence rate of 20–25% of the world’s adult population. Metabolic syndrome is often characterized by insulin resistance, which some have suggested is a major supportive connection between physical inactivity and MS. Various studies suggest that moderately elevated iron and ferritin levels are associated with an increased prevalence of metabolic syndrome and are markers of insulin resistance. Increased body iron stores are associated with the development of glucose intolerance, type 2 diabetes mellitus, and insulin resistance syndrome (IRS). This is a hospital-based cross-sectional observational study, which was conducted in the department of internal medicine of a tertiary care hospital in northern India. The study was conducted from 1 January 2019 to 30 June 2020 and included 100 patients and 100 controls. All subjects in the age group of 35–65 years were enrolled and investigated as per the study design. Metabolic syndrome patients were diagnosed according to the modified National Cholesterol Education Program Adult Treatment Panel-III (NCEP ATP-III) with BMI > 23 kg/m^2^. All baseline investigations were undertaken, including serum ferritin levels. Insulin resistance (IR) was calculated using the homeostasis model assessment IR (HOMA-IR) formula. We found a positive association between an increase in serum ferritin with the prevalence of metabolic syndrome and its clinical parameter. The serum ferritin level was positively correlated with the level of insulin resistance and inversely correlated with the insulin level of the patients.

## 1. Introduction

Metabolic syndrome (MS) is a group of conditions that includes central obesity, hypertriglyceridemia, low high-density lipoprotein (HDL) cholesterol, hyperglycemia, and hypertension and occurs in 20–25% of the world’s adult population [1]. Metabolic syndrome is also known as metabolic syndrome X, syndrome X, insulin resistance syndrome (IRS), Reaven’s syndrome, and CHAOS (coronary heart disease, hypertension, adult-onset diabetes, obesity, and stroke) [2]. Insulin resistance is characterized by a decrease in tissue sensitivity to the effects of insulin, resulting in a compensatory increase in insulin secretion. Several studies suggest that moderately elevated iron and ferritin levels are associated with an increased prevalence of metabolic syndrome and are markers of insulin resistance. Elevated endogenous iron stores are associated with the development of glucose intolerance, type 2 diabetes mellitus (T2DM), and insulin resistance syndrome (IRS). Iron overload leads to oxidative damage to pancreatic beta cells, and there is growing evidence that even moderately elevated iron stores represented by high-normal ferritin concentrations are associated with diabetes [3].

## 2. Aims and Objectives

To study serum ferritin as a confident marker in metabolic syndrome and its relationship with other components by measuring the blood levels of serum ferritin, fasting insulin, fasting blood glucose and post-prandial blood glucose, and lipid profile in metabolic syndrome patients and healthy controls.

## 3. Materials and Methods

The present study is a hospital-based cross-sectional observational study conducted in the department of general medicine of a tertiary care hospital in northern India. The study was conducted from 1 January 2019 to 30 June 2020. A total of 100 patients and 100 control subjects were enrolled in the study. All subjects aged 35–65 years who presented at the outpatient department (OPD) were included in the study and screened according to the study design. Patients with metabolic syndrome were diagnosed according to the modified National Cholesterol Education Programme Adult Treatment Panel-III (NCEP ATP-III) [4] with a body mass index (BMI) > 23 kg/m^2^. Those patients having cardiac, renal, hepatic, and other systemic diseases, any endocrinological abnormalities such as thyroid disorders, serum ferritin > 500 ng/mL, history of (h/o) blood transfusion, iron or vitamin therapies in the preceding six months, h/o blood donation in the preceding 4 months and h/o drug intake were excluded from the study.

For controls, inclusion criteria were a body mass index (BMI) of <23 kg/m^2^, haemoglobin > 12 gm/dL, FBS < 100 mg/dL, waist circumference for men < 90 and women < 80 centimetres, and a normal lipid profile and blood pressure. Each participant gave written informed consent to participate in the study. The study protocol was approved by the institutional review board and institutional ethics committee of the University with ref. no. SU/SMS&R/76-A/2018/126.

In this study, participants were divided into two groups based on the diagnostic criteria. Group I included cases of metabolic syndrome and group II comprised normal healthy controls. Various examinations were performed: Serum insulin level (by enzyme-linked immunoassay {ELISA}), fasting blood glucose and postprandial blood glucose (glucose oxidase method, Vitreos FS 5.2), fasting lipid profile (Vitreos FS 5.2), glycated haemoglobin (HbA1c) (Bio-Rad D10), complete blood count (CBC) (Sysmex Haematology analyzer XT-1800i), urinalysis (Urine Analyzer), and fasting serum ferritin level (semi-automated analyzer method). Insulin resistance (IR) was calculated using the homeostasis model assessment formula IR (HOMA-IR) [5], which is [fasting glucose level (mg/dL) × fasting insulin level (uIU/mL)/405]. If the value is >2.5, insulin resistance is present.

### Statistical Analysis

Results of continuous measurements are expressed as mean ± SD (min-max), and results of categorical measurements are expressed as number (%). Significance is assessed at a 5% significance level. The data are tested for normal distribution before the analysis of variance (ANOVA). ANOVA was used to determine the significance of study parameters between two or more patient groups; the chi-square/Fisher exact test was used to determine the significance of study parameters on a categorical scale with more than two groups. A 95% confidence interval was calculated to find the significant characteristics. A confidence interval with a lower bound of more than 50% is associated with statistical significance. A *p*-value of <0.05 was considered statistically significant. The Pearson correlation (r) was used to measure the linear correlation. It assigns a value between −1 and 1, where 0 is no correlation, 1 is a totally positive correlation, and −1 is a totally negative correlation.

## 4. Results

A total of 200 patients included in the present study were divided into two groups, 100 patients as cases with metabolic syndrome and 100 healthy individuals as controls. The mean age of the participants with metabolic syndrome was 57.35 ± 8.03 and that of the healthy controls was 56.71 ± 7.95, and the mean age difference between the groups was not statistically significant. In the present study, there were 128 male and 72 female participants, with males outnumbering females.

As shown in Table 1 BMI, waist circumference, systolic blood pressure, diastolic blood pressure, mean fasting blood glucose, postprandial blood glucose, mean cholesterol, serum triglycerides, serum LDL cholesterol, serum ferritin, serum insulin, and insulin resistance were significantly higher in metabolic syndrome cases compared with healthy control subjects. Mean haemoglobin and high-density cholesterol (HDL) were significantly lower in metabolic syndrome cases compared with healthy controls.

As shown in Table 2, the serum ferritin had a positive association with the serum level of glycated haemoglobin (HbA1c) (r = 0.185), the serum level of insulin (r = 0.546, *p* < 0.001), and insulin resistance (r = 0.512, *p* < 0.001). Similarly, insulin resistance showed a weak strength of association with the HbA1c level in patients (r = 0.094, *p* > 0.05). There was a strong positive significant association between insulin resistance and insulin levels in participants (r = 0.960, *p* < 0.001). Mean HbA1c was less than 6.5% in most of the cases of metabolic syndrome.

In the present study, after calculating the Pearson correlation of serum ferritin with the lipid profile, the serum ferritin had a significant positive strength of association of serum ferritin with the serum level of cholesterol (r = 0.391, *p* < 0.001), LDL-cholesterol (r = 0.601, *p* < 0.001), and triglycerides (r = 0.457, *p* < 0.001). However, the serum ferritin had a significant negative strength of association with serum HDL-cholesterol level (r = −0.594, *p* < 0.001). The insulin resistance also showed a significant positive strength of association with serum cholesterol (r = 0.359, *p* < 0.001), LDL-cholesterol (r = 0.512, *p* < 0.001), and triglycerides (r = 0.360, *p* < 0.001). Insulin resistance showed a negative strength of association with the HDL-cholesterol level (r = −0.256, *p* < 0.001), as shown in Table 3.

### Components of Metabolic Syndrome

There were 35 (35%) patients with three components of metabolic syndrome, 37 (37%) with four components, and 28 (28%) with five components of metabolic syndrome

As shown in Table 4, the individual components of the metabolic syndrome had a significant difference with an increasing number of components of metabolic syndrome.

## 5. Discussion

Chronic inflammation is commonly associated with metabolic syndrome. Inflammatory markers that have been associated with metabolic syndrome are hs-CRP, TNF-α, fibrinogen, and IL-6 [6]. These proteins are elevated in the blood when a subclinical inflammatory process is present. Serum ferritin is an acute-phase reactant, it may be elevated in the presence of inflammation. The study by Liu JR et al. [7] showed that serum ferritin is an early marker of cardiovascular risk. In our present study, serum ferritin was significantly elevated in metabolic syndrome case at 224.04 ± 53.12 compared with healthy controls at 68.9 ± 56 (*p*-value < 0.001) (Table 1). There is also a significant positive association between serum ferritin with insulin, insulin resistance, serum cholesterol, LDL, and triglycerides, and a negative association with serum HDL (*p*-value < 0.001) (Table 2 and Table 3). J Shivasankari et al. [8], Wang M. et al. [9], Cho M.R. et al. [10] and other similar studies have shown that serum ferritin, iron, insulin, and HOMA-IR are significantly increased in metabolic syndrome compared to healthy controls (*p* < 0.001).

Also, in our study, most patients were between 40 and 50 years old, and this suggests that IR begins to develop in the fourth decade of life along with the development of hypertension, dyslipidaemia, and obesity. These physical characteristics were significantly higher in metabolic syndrome patients compared with healthy controls (Table 1). In a similar study, Momeni A et al. [11] showed that the mean age of patients was 56.5 ± 9.7 (30 to 82) years, and serum ferritin decreased significantly after the control of hyperglycaemia.

We also found that haemoglobin level was significantly lower in metabolic syndrome patients compared with healthy controls. Peslova G et al. [12] and Nemeth E et al. [13] also demonstrated lower haemoglobin levels in cases compared with controls.

In patients with metabolic syndrome, mean fasting blood glucose (117.12 mg/dL) and mean postprandial blood glucose (184.04 mg/dL) were statistically higher than in the healthy control group patients, who had mean fasting blood glucose of 98.05 mg/dL and mean postprandial blood glucose of 130.41 mg/dL. There is also a significant relationship between ferritin and glycaemic control (Table 2). Some studies such as Raj S et al. [14], Maiti T et al. [15], and Gupta A et al. [16] also showed similar results. The lipid profile of the patients was significantly different from that of the healthy control group. Mean serum total cholesterol (229.82 ± 45.08), LDL cholesterol (128.59 ± 32.54), and triglycerides (161.86 ± 27.11) were significantly higher in metabolic syndrome cases compared with normal healthy controls. Howard B V [17], Hoenig MR et al. [18], and other studies showed similar results. Serum levels of insulin were significantly higher in patients with metabolic syndrome (56.14 ± 6.75) than in normal controls (10.91 ± 2.12). Insulin resistance was statistically significantly higher in metabolic syndrome cases (13.30 ± 1.98) than in healthy control subjects (4.31 ± 1.59) (Table 1). Sudhakar B et al. [19], Padwal MK et al. [20], and other similar studies showed a significant increase in serum insulin levels and insulin resistance in metabolic syndrome cases.

In our study, we also found that the most common three components (35%) were fasting plasma glucose ≥ 110 mg/dL (approximately 85%), plus BP ≥ 130/85 mmHg (approximately 71%), plus central obesity: waist circumference ≥ 102 cm in men and ≥88 cm in women (approx.48%); the most common four components (37%) were fasting plasma glucose ≥ 110 mg/dL (approx.87%), plus BP ≥ 130/85 mmHg (approx. 84%), plus central obesity: waist circumference ≥ 102 cm in men and ≥88 cm in women (approx. 84%), plus dyslipidemia: HDL < 40 mg/dL (male) and <50 mg/dL (female) (approximately 76%), and all five components were present in the 28% of metabolic syndrome cases. In a similar study, Shim YS et al. [21] also showed that patients with metabolic syndrome had significantly higher BMI, waist and hip circumferences, systolic and diastolic pressures, fasting glycemia, 2 h postprandial serum glucose, total cholesterol, triglycerides, and lower HDL cholesterol. Individual components of the metabolic syndrome showed a significant difference with increasing numbers of metabolic syndrome components (Table 4). Central obesity was associated with an increasing number of metabolic syndrome components with a significant *p*-value of <0.001, and dyslipidemia (both triglycerides and HDL criteria) also showed a similar correlation of statistical significance (*p*-value of <0.001). Blood pressure was also significantly correlated with a *p*-value of 0.003, and blood glucose showed no statistical significance (*p* = 0.079+). The present study also shows a positive correlation between the altered lipid profiles, insulin, and insulin resistance in patients with metabolic syndrome.

## 6. Limitations

The major limitation of our study is that it is an observational cross-sectional study, and we cannot find any evidence of a temporal relationship between exposure and outcomes. Additionally, we did not include patients who were on drugs as this may have altered the serum ferritin levels.

## 7. Conclusions

In our study, we found a positive association between an increase in serum ferritin and the prevalence of metabolic syndrome and its clinical parameters. Serum ferritin was positively correlated with the degree of insulin resistance and inversely correlated with insulin levels in patients with metabolic syndrome. Ferritin was also strongly positively correlated with elevated cholesterol, LDL, and triglyceride levels and negatively correlated with HDL levels. There is also a significant correlation between the individual components of the metabolic syndrome and elevated serum ferritin.

## Figures and Tables

**Table 1:** Mean levels of body mass index (BMI), waist circumference, blood pressure, haemoglobin level (Hb), fasting and postprandial blood sugar, lipid profile, serum ferritin, serum insulin, and insulin resistance.

	Metabolic Syndrome (Cases)		Healthy Control		*p*-Value *
	Mean	SD	Mean	SD	
BMI in kg/m^2^	29.43	1.761	22.10	2.01	<0.001 *
Waist circumference in centimetres	99.05	6.866	82.09	7.12	<0.001*
Systolic BP in mmHg	146.62	14.603	115.6	12.19	<0.001 *
Diastolic BP in mmHg	92.76	8.574	76.79	7.51	<0.001*
Hb in g/dL	13.9	0.95	14.35	1.05	<0.001 *
Fasting blood sugar in mg/dL	117.12	8.19	98.05	12.15	<0.001*
Post prandial blood sugar in mg/dL	184.04	12.33	130.41	16.22	<0.001 *
Total cholesterol in mg/dL	229.82	45.08	185.04	26.89	<0.001*
Triglyceride in mg/dL	161.86	27.114	132.49	20.28	<0.001 *
HDL Cholesterol in mg/dL	32.87	5.691	46.17	4.61	<0.001 *
LDL cholesterol in mg/dL	128.59	32.54	110.26	22.15	<0.001 *
Ferritin in ng/mL	224.04	53.12	68.9	25	<0.001 *
Serum Insulin mIU/L	56.14	6.75	10.91	2.12	<0.001 *
Insulin Resistance	13.30	1.98	4.31	1.59	<0.001 *

Statistically significant * (*p*-value = 0.000000000023).

**Table 2:** Showing Pearson correlation of serum ferritin with HbA1c, insulin and insulin resistance.

		Glycated Haemoglobin (HbA1c)	Insulin	Insulin Resistance
Glycated Haemoglobin (HbA1c)	Pearson Correlation		0.130	0.094
	Sig. (2-tailed)		0.192	0.350
Insulin Resistance	Pearson Correlation	0.094	0.960 **	
	Sig. (2-tailed)	0.350	0.000	
Ferritin	Pearson Correlation	0.185	0.546 **	0.512 **
	Sig. (2-tailed)	0.063	0.000	0.000

** Correlation is significant at the 0.01 level (2-tailed).

**Table 3:** Pearson correlation of the insulin resistance and serum ferritin with other parameters.

		Insulin Resistance	Serum Ferritin
Cholesterol	R	0.359 **	0.391 **
	Sig	0.000	0.000
TG	R	0.360 **	0.457 **
	Sig	0.000	0.000
LDL	R	0.512 **	0.601 **
	Sig	0.000	0.000
HDL	R	−0.256 **	−0.594 **
	Sig	0.000	0.000

** Correlation is significant at the 0.01 level (2-tailed).

**Table 4:** Correlation of components of metabolic syndrome with severity.

Variables	The Severity of Metabolic Syndrome			*p*-Value
	3 Components (*n* = 35)	4 Components (*n* = 37)	5 Components (*n* = 28)	
central obesity: waist circumference ≥ 102 cm in male ≥ 88 cm in female	17 (48.6%)	31 (83.7%)	28 (100%)	<0.001 **
dyslipidaemia: TG ≥ 150 mg/dL	16 (45.7%)	26 (68.4%)	28 (100%)	<0.001 **
dyslipidaemia: HDL-C < 40 mg/dL (male), <50 mg/dL (female)	16 (45.7%)	28 (75.7%)	28 (100%)	<0.001 **
blood pressure ≥ 130/85 mmHg	25 (71.4%)	32 (84.2%)	28 (100%	0.003 **
fasting plasma glucose ≥ 110 mg/dL	30 (85.7%)	33 (86.8%)	28 (100%)	0.079+

Statistically significant ** (*p*-value = 0.000000000023). Central obesity was associated with an increasing number of components of metabolic syndrome with a significant *p*-value of <0.001, and dyslipidaemia (both triglycerides and HDL criteria) also showed a similar correlation of statistical significance (*p*-value of <0.001). Blood pressure was also significantly correlated with a *p*-value of 0.003, and blood sugars did not show statistical significance (*p* = 0.079+).

## Data Availability

Not applicable.
